# Factor inhibiting HIF1–A novel target of SUMOylation in the human placenta

**DOI:** 10.18632/oncotarget.23113

**Published:** 2017-12-07

**Authors:** Julien Sallais, Sruthi Alahari, Andrea Tagliaferro, Jayonta Bhattacharjee, Martin Post, Isabella Caniggia

**Affiliations:** ^1^ Lunenfeld-Tanenbaum Research Institute, Mount Sinai Hospital, Toronto, Ontario, Canada; ^2^ Department of Obstetrics and Gynaecology, University of Toronto, Ontario, Canada; ^3^ Department of Physiology, University of Toronto, Ontario, Canada; ^4^ Institute of Medical Sciences University of Toronto, Toronto, Ontario, Canada; ^5^ Program in Physiology and Experimental Medicine, Peter Gilgan Centre for Research and Learning, Hospital for Sick Children, Toronto, Ontario, Canada

**Keywords:** factor Inhibiting HIF, hypoxia-inducible factor, SUMOylation, placenta, preeclampsia

## Abstract

Adaptations to changes in oxygen are critical to ensure proper placental development, and impairments in oxygen sensing mechanisms characterize placental pathologies such as preeclampsia. In this study, we examined the involvement of SUMOylation, a reversible posttranslational modification, in the regulation of the asparaginyl hydroxylase Factor Inhibiting Hypoxia Inducible Factor 1 (FIH1) in the human placenta in development and in disease status. FIH1 protein abundance and spatial distribution in the developing placenta directly correlated with oxygen tension *in vivo*. Immunofluorescence analysis showed that early on FIH1 primarily localized to nuclei of cytotrophoblast cells, while after 10 weeks of gestation it was present in nuclei and cytoplasm of both cytotrophoblast and syncytiotrophoblast cells. Exposure of choriocarcinoma JEG-3 cells to hypoxia induced FIH1 SUMOylation by promoting its association to SUMO2/3. Transfection of JEG-3 cells with FIH1 constructs containing SUMO-mutated sites revealed that SUMOylation of FIH1 by SUMO2/3 targeted it for proteasomal degradation, particularly in hypoxia. SUMOylation of FIH1 directly impacted on HIF1A activity as determined by HIF-responsive luciferase assay. Co-immunoprecipitation analyses revealed enhanced FIH1-SUMO2/3 associations early in development, when FIH1 levels are low, while deSUMOylation of FIH1 by SENP3 increased later in gestation, when FIH1 levels are rising. In preeclampsia, decreased FIH1 protein expression associated with impaired deSUMOylation by SENP3 and increased association with the ubiquitin ligase RNF4. We propose a novel mode of regulation of FIH1 stability by dynamic SUMOylation and deSUMOylation in the human placenta in response to changing oxygen tension, thereby mediating HIF1A transcriptional activity in physiological and pathological conditions.

## INTRODUCTION

During early placental development changes in oxygen tension guide proper trophoblast differentiation events and disruption in oxygen sensing has been shown to lead to preeclampsia (PE) [1, 2]. PE is a complex disease of the placenta that affects 5–7% of pregnancies worldwide and clinically manifests as maternal hypertension and either proteinuria, or end organ damage [3]. At the molecular level, one of the defining features of this pathology is the high expression of the highly conserved Hypoxia-Inducible Factor 1α (HIF1A) [4], a key oxygen sensor that mediates the global transcriptional response to hypoxia [5]. HIF1 elicits its function as a heterodimer composed of a constitutively expressed HIF1B subunit and one of three oxygen-inducible subunits, HIF1A, HIF2A or HIF3A [4]. In hypoxia, HIF1 heterodimerization leads to its binding to conserved hypoxia responsive elements (HRE), located in the promoter of hypoxia-responsive target genes. Activation of a global physiological response to hypoxia typically triggers the transcription of genes involved in angiogenesis, cell fate and anaerobic metabolism, thereby maintaining cell homeostasis [6, 7]. HIF1A protein stability is tightly regulated in normoxic conditions by prolyl hydroxylase (PHD) enzymes, namely PHD1, PHD2 and PHD3 [8]. Hydroxylation on two specific proline residues (402 and 564) on the oxygen-dependent degradation domain (ODD) of HIF1A allows the recruitment of the von Hippel Lindau (VHL) [9] tumor suppressor protein that subsequently targets HIF1A for rapid proteasomal degradation. In addition, another hydroxylase, named Factor Inhibiting HIF1 (FIH1), regulates the transcriptional activity of HIF1A [10]. In association with VHL, FIH1 targets and hydroxylates a specific asparaginyl (803) residue located in the C-TAD domain of HIF1A, thereby disrupting HIF1A binding to its transcriptional co-activator, CBP/p300 [11] and repressing HIF1A transcriptional activity. In hypoxia, FIH1 activity is inhibited and HIF1A is free to be transcriptionally active [10].

The expression of prolyl hydroxylases is oxygen-dependent [1, 12, 13] and their protein stability is in part reliant on the action of specific E3-ubiquitin ligases, termed SIAHs, which target the PHDs for proteasomal degradation [14]. While studies have established a role for miRNAs (miR-31 and miR-135b) and the chromatin remodeler ISWI (Imitating SWI) in regulating FIH1 mRNA expression [15, 16], the precise mechanisms controlling FIH1 protein stability are unknown.

SUMOylation is a reversible posttranslational modification event that regulates protein stability and activity in eukaryotic cells [17]. The Small Ubiquitin-related Modifier (SUMO) family is composed of four members, namely SUMO1, SUMO2, SUMO3 and SUMO4. Since the protein sequence identity between SUMO2 and SUMO3 is 97%, they are collectively referred to as SUMO2/3 [18]. SUMOylation is a dynamic and rapid process that involves SUMO conjugation to the target protein followed by its deSUMOylation [19], which is executed by a family of Sentrin-specific proteases (SENPs) [20]. Typically, SENP proteins show specificity not only for their protein substrates, but also for particular SUMOs [19]. For instance, SENP3 protease has been described to preferentially interact with SUMO2/3 [21].

The SUMOylation machinery shares similarities with the ubiquitin-ligase system, not only structurally but also in the mode of enzymatic processing of substrate proteins *via* the formation of covalent bonds [17]. Moreover, similar to ubiquitination, the functional consequences of protein SUMOylation are diverse and target-specific. Three main functional events associated with SUMOylation include: 1) interference of binding between the target and its cofactor by masking the surface of interaction, 2) induction of a conformational change of the target protein, and 3) generation of a SUMO-dependent interaction with downstream effectors [20]. The latter consequence of SUMOylation can lead to the recruitment of specific ubiquitin ligases to guide the target SUMOylated protein to proteasomal degradation [22]. Indeed, several E3-ubiquitin ligases interact specifically with SUMO conjugates, and are aptly termed SUMO-targeted ubiquitin ligases (STUbL) [23, 24]. In particular, the RING finger protein 4 (RNF4) described initially in promyelocytic leukemia cells (PML), has been shown to target a variety of SUMO2/3 conjugates for proteasomal degradation [25].

Originally demonstrated in a model of cerebral ischemia, hypoxia is reported to induce a global level of protein SUMOylation [26]. Interestingly, mounting evidence indicates that key players in the cellular oxygen-sensing pathway are themselves subject to SUMOylation [27]. SUMOylation of VHL is important for its stabilization and nuclear sequestration, thereby impacting on HIF1A ubiquitination and proteasomal degradation [28]. In hypoxic conditions, VHL is capable of targeting SUMOylated HIF1A for degradation [29]. By contrast, H_2_O_2_-induced stress triggers p300 deSUMOylation by SENP3, thereby stimulating HIF1A activity in HeLa cells [30]. SUMOylation of PHD3 contributes to PHD3-mediated repression of HIF1A [29, 31], and we recently showed that in conditions of low oxygen, HIF1A is targeted for degradation by SUMO2/3 in the human placenta [32].

Although hypoxia regulates PHD gene expression, FIH1 transcription is not directly controlled by O_2_ [1]. However, in the developing placenta, changes in FIH1 protein are consistent with an O_2_-dependent regulation [1]. Hence, we investigated whether SUMOylation of FIH1 regulated its stability in the developing human placenta. We report for the first time that under hypoxic conditions, FIH1 is subject to SUMO2/3 conjugation, which in turn leads to its proteasomal degradation, suggesting that during human placental development, FIH1 SUMOylation maintains cellular homeostasis by tightly regulating HIF1A activity.

## RESULTS

### FIH1 temporal and spatial expression patterns coincide with variations in oxygen tension

**Figure 1:**
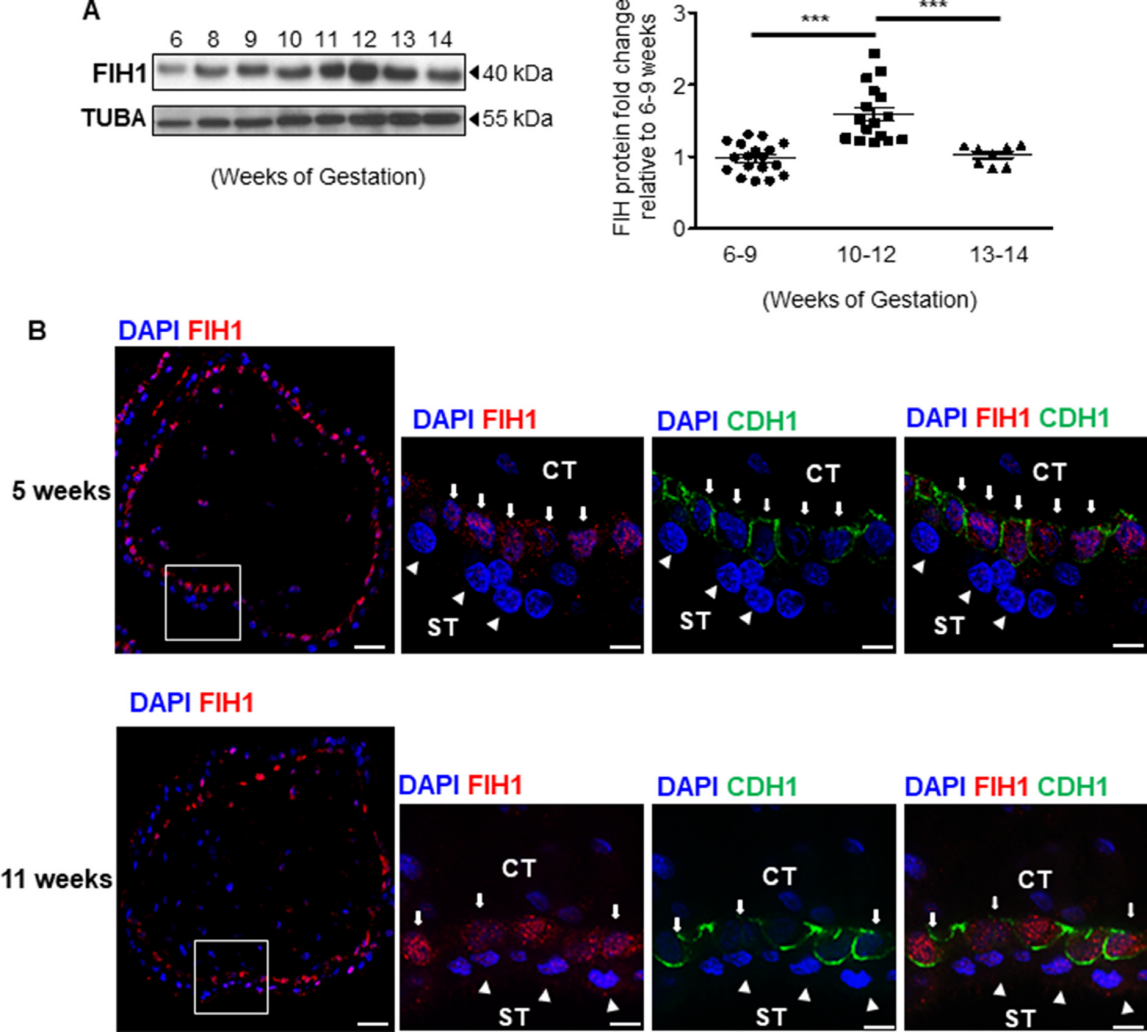
Expression of FIH1 during early placental development (**A**) Representative Western Blot of FIH1 expression in first trimester placental tissue (left panel). Right Panel: Densitometric analysis of FIH1 protein expression in early (5-9 weeks, *n* = 18) and late (10-12 weeks, *n* = 17 ; 13-14 weeks, *n* = 7) first trimester placental tissues (right panel, ^*^p < 0.0001, Mann-Whitney *U*-test). ACTB was used as loading control. Values are mean ± SEM. (**B**) Spatial localization of FIH1 (red) and E-cadherin (green) in placental section from 5 and 11 weeks gestation. Nuclei were counterstained with DAPI (blue). (CT: cytotrophoblast cells indicated by arrows; ST: syncytiotrophoblast cells indicated by arrowheads); White bars represent 50 µm at lower magnification and 25µm at higher magnification.

We have previously reported that in the developing placenta, FIH1 mRNA peaks at the end of the first trimester of gestation, when oxygen tension rises and HIF1A expression decreases [13]. Hence, we first examined the spatio-temporal distribution of FIH1 protein in placental tissue during early gestation. In line with our previous FIH1 transcript findings [13], FIH1 protein levels peaked at 10–12 weeks and declined thereafter (Figure 1A). Goat anti-human FIH1 specificity was validated using a synthetic competing peptide. The antibody detected a 40kDa band, the expected molecular weight of FIH1 [1]; which was effectively competed by the peptide (Supplementary Figure 1A).

**Figure 2:**
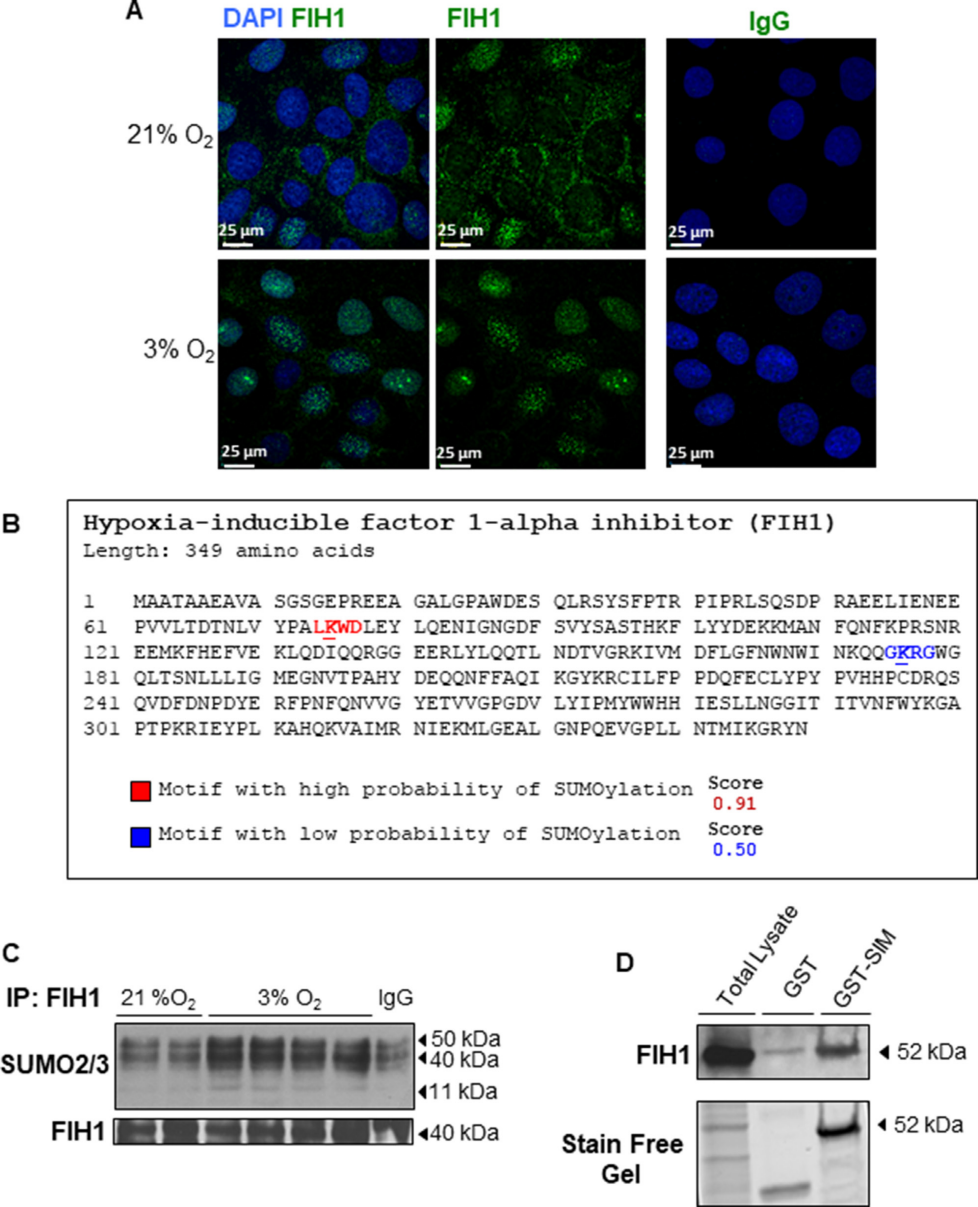
Localization and post-translational modification of FIH1 in hypoxia (**A**) Spatial localization of FIH1 (green) in JEG-3 cells incubated in 3% O_2_ or standard conditions. Nuclei were counterstained with DAPI (blue). White bars represent 25 µm. (**B**) *In silico* analysis of FIH1 protein sequence indicating potential SUMOylation sites with the high probability motif (red) and the low probability motif (blue). Analysis was performed using SUMOplot™ analysis program. (**C**) Representative Western Blots showing SUMO conjugation of FIH1 by SUMO2/3 in JEG3-cells exposed to hypoxic or standard conditions. (**D**) Western Blot for FIH1 before and after enrichment of polySUMOylated protein in whole JEG-3 cells lysate. Total: Total Cell Lysate; GST: Control affinity purification; GST-SIM: Affinity Purification of PolySUMOylated Protein. Protein loading was assessed using stain free activation of the gel.

We next examined the spatial localization of FIH1 in placental sections across the first trimester of gestation. Immunofluorescence analysis showed positive nuclear FIH1 signal primarily in cytotrophoblast cells of chorionic villi in placental sections from 5 weeks gestation (Figure 1B top panel). Interestingly, at 11 weeks of gestation, nuclear and cytoplasmic FIH1 signal was detected in both cytotrophoblast and syncytiotrophoblast cells of chorionic villi (Figure 1B bottom panel). Staining for E-Cadherin (CDH1), a marker of cytotrophoblastic cells, confirmed FIH1 expression in these cells (Figure 1B). Owing to its function as an oxygen sensor, we further examined whether FIH1 expression and spatial distribution was dependent on changes in oxygen tension. We employed an established *in vitro* model of JEG-3 choriocarcinoma cells, which were exposed to varying oxygen tensions. No changes in FIH1 protein abundance were found between 3% *versus* 21% O_2_ exposure (data not shown). However, immunofluorescence analysis demonstrated that after exposure of JEG-3 cells to 3% O_2_, FIH1 , had a marked nuclear appearance when compared to cells exposed to 21% O_2_ (Figure 2A), in line with the prominent nuclear localization of FIH1 in placental sections at 5 weeks gestation when pO_2_ is low (Figure 1B).

### FIH1 is targeted for SUMOylation in hypoxia

SUMO modification of proteins that are central to the hypoxia-signalling cascade is critical for the cellular adaptive response to hypoxia/oxidative stress [27], and we have recently reported that HIF1A is a target of SUMOylation in the human placenta [32]. Given the lack of understanding on the precise mechanisms regulating FIH1 stability, we investigated whether FIH1 is targeted for SUMOylation. SUMOylation occurs via the formation of a covalent bound between a lysine (K) of the targeted protein and the C-carboxyl terminal domain of SUMO proteins. The K of the protein substrate is contained in a specific consensus sequence, namely ψ-K-X-E (ψ is an aliphatic amino acid, X is any residue and E is an acidic residue). It is possible to detect consensus SUMOylation sites within the sequence of a protein via *in silico* analysis.

**Figure 3:**
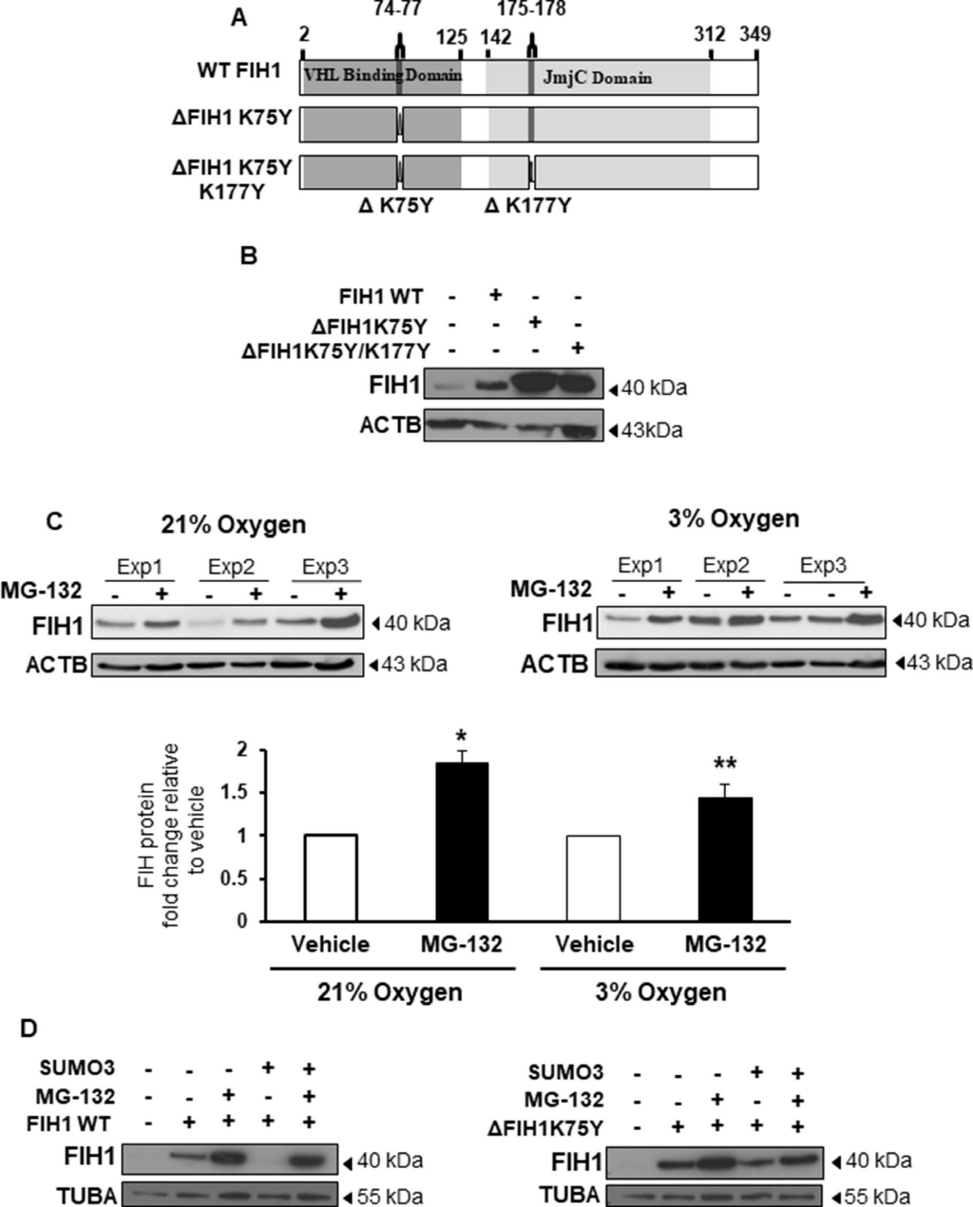
SUMOylation of FIH1 targets it for proteasomal degradation (**A**) Schematic representation of FIH1 protein wild type (WT) construct containing a VHL Binding Domain (dark grey), a JmjC domain (light grey), high probability (ΔK75Y) and low probability (ΔK177Y) SUMOylation sites. Mutated FIH1 constructs (ΔK75Y and ΔK75YΔK177Y respectively) were generated by single-point mutations. (**B**) Representative Western Blots of FIH1 following FIH1 overexpression using plasmids constructs containing either FIH1 WT, FIH1 ΔK75Y, or FIH1 ΔK75Y ΔK177Y. (**C**) Top panel: representative Western Blot of FIH1 in JEG-3 cells following treatment with the proteasome inhibitor (MG-132) in 21% (left) or 3% oxygen (right). Bottom panel: Densitometric analysis of FIH1 protein expression in MG-132 treated JEG-3 cells. Data are expressed as a fold change relative to control (V) vehicle (21% oxygen: *n* = 5, ^*^p < 0.05, Mann-Whitney Test; 3% oxygen *n* = 5, ^**^p < 0.01, Mann-Whitney Test). (**D**) Representative Western Blots for FIH1 in JEG-3 following overexpression of FIH1 WT (left panel) or FIH1 ΔK75Y (right panel) and SUMO3 protein in the presence and absence of the MG-132.

Using SUMOplot™ analysis program (http://www.abgent.com/sumoplot), which predicts the probability of a consensus SUMO sequence within a protein to be subject to SUMOYlation, we conducted *in silico* analysis and detected two potential sites for SUMOylation: a high probability site spanning the VHL binding domain of FIH1 and a low probability site spanning the Jumonji C (JmjC) domain of FIH1 (Figures 2B and 3A). Immunoprecipitation of FIH1 followed by immunoblotting for SUMO2/3 showed an increased SUMO2/3 and FIH1 association in JEG-3 cells cultured at 3% O_2_ versus 21% O_2_. SUMO2/3-FIH1 conjugates are located around 40–52 kDa, as the denaturing condition of the IP should not disrupt the covalent bound formed between SUMO and the substrate protein. We also detected increased free SUMO2/3 at 11 kDa in 3% oxygen (Figure 2C), suggesting that FIH1 may be targeted for SUMOylation, particularly in conditions of reduced oxygenation. To conclusively confirm that FIH1 is a target of SUMOylation, we used a commercially available capture & release kit that permits the enrichment of polySUMOylated proteins in cell lysates. This kit takes advantage of SIM domains contained in the RNF4 protein to specifically capture polySUMO chain-associated proteins. Following elution of polySUMO-enriched JEG-3 lysates, WB analysis for FIH1 revealed increased FIH1 levels in the elution from the GST-associated with the SIM domains (GST-SIM) fraction of when compared to the negative control GST alone (Figure 2D). These data demonstrate that FIH1 is indeed a target of SUMOylation in JEG-3 cells kept at 3% O_2_.

### SUMOylation brings FIH1 to degradation

Having established that FIH1 is a target of SUMO2/3 in low oxygen conditions, we next investigated the functional consequence of this modification. Hence, we generated pcDNA3.1 expression constructs carrying missense mutations in either the high probability SUMOylation site (designated ΔFIH1_K75Y_) or in both predicted SUMOylation sites (designated ΔFIH1_K75Y/K177Y_), and transiently transfected them into JEG-3 cells (Figure 3A). FIH1 WT and empty vector constructs were used as controls. Western Blotting for FIH1 following 48 hours of transfection with the three FIH1 constructs revealed an increase in FIH1 protein abundance relative to control empty vector (Figure 3B). Notably, transfection with either ΔFIH1_K75Y_ or ΔFIH1_K75Y/K177Y_ resulted in a further increase in FIH1 levels relative to FIH1_WT_ transfected cells (Figure 3B). Transfection with ΔFIH1_K75Y/K177Y_ caused a similar increase in FIH1 as transfection with ΔFIH1_K75Y,_ suggesting that the K75Y mutation was responsible for the increase in FIH1 and, therefore, further experiments were performed with this construct only. To account for potential epitope changes resulting from mutation-induced conformational changes, we confirmed the FIH1 results with another FIH1 antibody that recognizes an epitope (C-terminus of FIH1) outside of the sequence affected by the mutations (Supplementary Figure 1B). Taken together, these data indicate that SUMOylation of FIH1 may play a role in controlling FIH1 stability. Since canonical protein degradation typically occurs through the ubiquitin-proteasome pathway, we examined whether SUMOylation of FIH1 impacted on its proteasomal degradation. Exposure of JEG-3 cells to MG-132, a selective inhibitor of the 26S proteasome [33], revealed a significant increase in FIH1 protein levels relative to vehicle-treated controls in cells maintained at both 21 and 3% oxygen (Figure 3C). To further examine whether this was dependent on FIH1 SUMOylation, and to precisely identify the SUMO protein responsible for FIH1 destabilization, we co-transfected FIH1_WT_ and ΔFIH1_K75Y_ constructs with either SUMO1, SUMO2, or SUMO3 expression vectors in JEG-3 cells in the presence or absence of MG-132. For proper comparison within each different SUMO species overexpression, WB for FIH1 were processed, probed and developed at the same time using the same experimental conditions; and each SUMOs overexpression was validated by Western blotting (Supplementary Figure 1C). As anticipated, FIH1 overexpression, either WT or mutant, alone or in combination with the proteasome inhibitor, increased FIH1 levels relative to untreated controls (Figure 3D and Supplementary Figure 1D and 1E). Of note, while co-transfection of FIH1 with each of the SUMOs decreased FIH1 protein abundance, the most robust effect was obtained following transfection with SUMO3, showing the total disappearance of the FIH1 band following co-transfection of FIH1_WT_ with SUMO3 (Figure 3D bottom panels). By contrast, FIH1 levels remained constant when ΔFIH1_K75Y_ was co-transfected with SUMO2/3 (Figure 3D, right panel. Moreover, addition of MG-132 largely rescued SUMO-mediated destabilization of FIH1 (Figure 3D, left panel), suggesting that SUMOylation of FIH1, particularly by SUMO3, promotes its proteasomal degradation.

### FIH1 SUMOylation in hypoxia enhances HIF1A transcriptional activity

**Figure 4:**
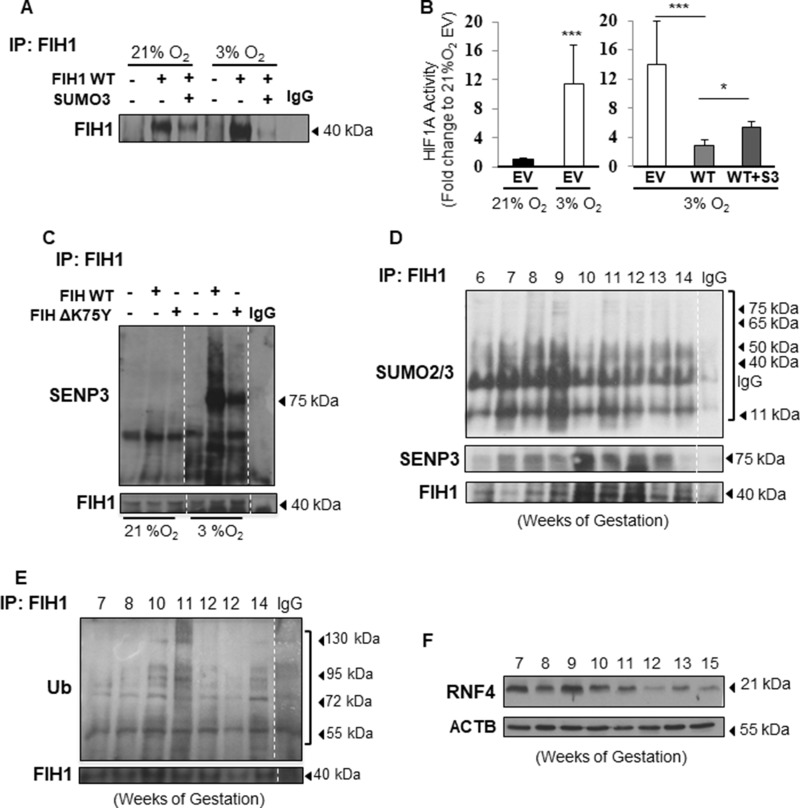
FIH1 SUMOylation and deSUMOylation are enhanced in hypoxia (**A**) Representative Western Blots of FIH1 following immunoprecipitation of FIH1 in HEK293 cells upon overexpression of FIH1 and SUMO3 in 3% O_2_ and standard conditions (21% O_2_). (**B**) Luciferase reporter assay showing HIF1A activity in HEK293 cells following overexpression of FIH1 WT alone or in combination with SUMO2/3. EV = Empty Vector, WT = FIH1 wild type, WT + S3 = FIH1 WT with SUMO2/3. *N* = 4, *p* < 0.001 (21% EV vs. 3% EV), *p* < 0.001 (21% EV vs. 3% EV) One-way ANOVA; *p* = 0.04 (3% WT vs. 3% WT+S3), Student’s *T*-test and Mann-Whitney post-test. (**C**) Representative Western Blots for SENP3, SUMO2/3 and FIH1 following immunoprecipitation of FIH1 in JEG-3 cells incubated in 3% O_2_ or 21% O_2_ following overexpression of FIH1 WT or ΔK75Y mutant. (**D**) Representative Western Blots for SUMO2/3, SENP3 and FIH1 after FIH1 immunoprecipitation during early placental development. (**E**) Western Blot for ubiquitin (Ub) and FIH1 following immunoprecipitation of FIH1 in early gestation. (**F**) Western Blot for RNF4 in early placental development.

Given that FIH1 regulation has significant consequences for HIF1A signalling, we next sought to examine the impact of FIH1 SUMOylation on its protein stability under different oxygen tension conditions. Western blotting for FIH1 following immunoprecipitation of FIH1 in cells overexpressing SUMO2/3 and kept at either 3% or 21% O_2_, revealed that the reduction in FIH1 stability by SUMO2/3 was more pronounced in JEG-3 cells exposed to 3% O_2_ (Figure 4A). This prompted us to examine whether SUMOylated FIH1 affected downstream HIF1A transcriptional activity, particularly in hypoxia. Hence, we employed an HRE-luciferase gene reporter system as readout for HIF1A activity in hypoxia. As anticipated, HIF1A activity was significantly higher in JEG-3 cells cultured at 3% O_2_ than 21% O_2_, suggesting that the HRE system was responding to the variation in oxygen (Figure 4B, left panel). Importantly, overexpression of wild type FIH1 in JEG-3 cells maintained at 3% O_2_ significantly reduced HIF1A activity to levels equivalent to baseline normoxic activity, while overexpression of SUMO2/3 in conjunction with WT FIH1 increased HIF1A activity relative to wild type FIH1 alone (Figure 4B, right panel).

### FIH1 is targeted for deSUMOylation by SENP3

SUMOylation is a rapid process that is reversed by Sentrin-specific proteases, enzymes responsible for removal of the SUMO protein from its target. This process is referred to as deSUMOylation and is a physiological complement to SUMOylation of proteins [34]. To uncover the contribution of deSUMOylation in modulating FIH1 stability, we overexpressed either FIH1_WT_ or ΔFIH1_K75Y_ in JEG-3 cells. Immunoprecipitation of FIH1 followed by SENP3 immunoblotting revealed an overall augmented deSUMOylation of FIH1 at 3% O_2_ relative to 21% O_2_ (Figure 4C). When FIH1_WT_ was overexpressed, SENP3 associated to FIH1 (75 kDa band) in JEG-3 cells cultured at 3% O_2,_ but not in cells maintained at 21% O_2_, which correlates with the increased FIH1-SUMO2/3 conjugates_._ However, SENP3-FIH1 and SUMO2/3-FIH1 association at 3% O_2_ was reduced upon overexpression of FIH1_ΔK75Y_ (Figure 4C). This show that overexpression of the mutated FIH1 constructed FIH1_ΔK75Y_ disrupt binding between FIH1 and SUMO2/3 and suggests that FIH SUMOylation is tightly regulated in hypoxia and compensated by deSUMOylation in order to maintain FIH1 homeostasis.

**Figure 5:**
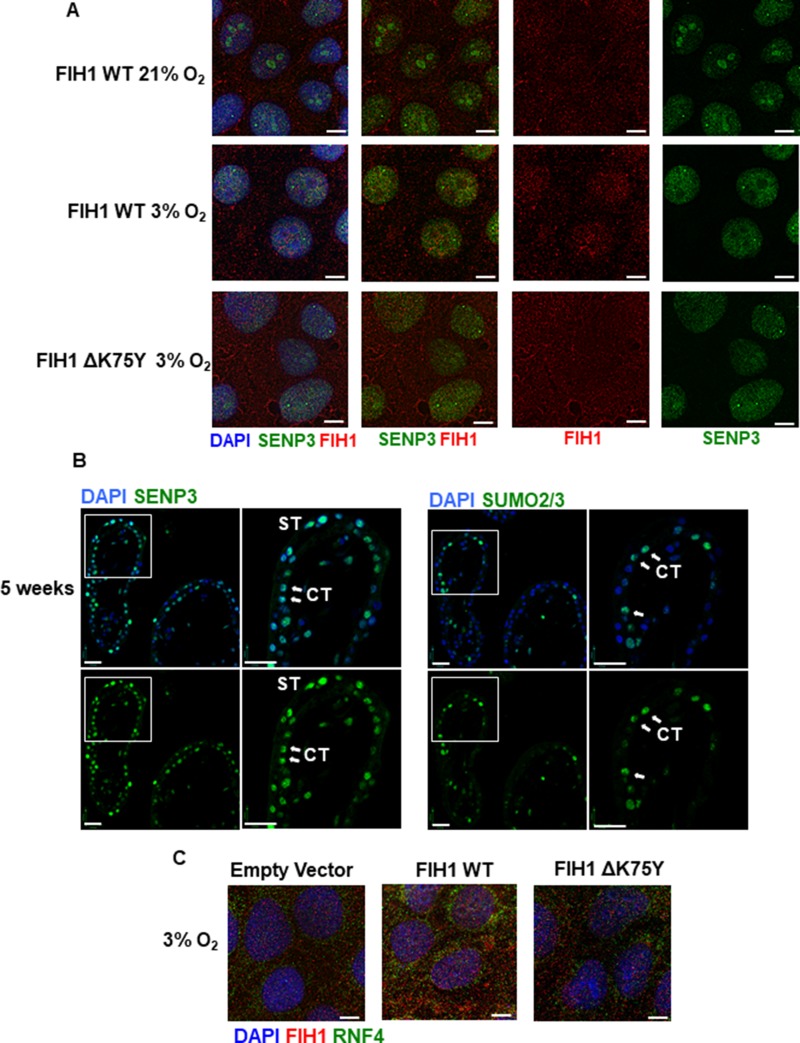
FIH1 deSUMOylation and association to RNF4 in JEG-3 cells (**A**) Immunofluorescence analysis of FIH1 (red) and SENP3 (green) in JEG-3 cells following overexpression of FIH1 WT or ΔK75Y mutant in 3% O_2_ or standard conditions (21% O_2_). Nuclei were counterstained with DAPI (blue). White bars represent 25 µm. (**B**) Spatial localization of SENP3 (left panels) and SUMO2/3 (right panels) in early first trimester placental tissue sections (5 weeks). Nuclei were counterstained with DAPI (blue). White bars represent 50 µm. (**C**) Immunofluorescence analysis of FIH1 (red) and RNF4 (green) in JEG-3 cells upon overexpression of FIH1 WT or ΔK75Y at 3% O_2_. Nuclei were counterstained with DAPI (blue). White bars represent 25 µm.

We have recently reported that placental SENP3 is sequestered to the nucleoli of JEG-3 cells in ambient air and, upon exposure to hypoxia, relocates to the nucleoplasm where it becomes active [32]. To visualize potential SENP3-FIH1 interactions in hypoxia, we transfected JEG-3 cells at ambient air with FIH1_WT_ and ΔFIH1_K75Y_ expressing vectors, exposed them to 3% or 21 % O_2_ for 24 hours and then assessed SENP3 and FIH1 cellular distribution. In agreement with our previous findings, in normoxia, SENP3 spatial localization was restricted to the nucleoli, while FIH1 was predominantly cytoplasmic (Figure 5A top panels). Exposure of cells to 3% O_2_ resulted in a nucleoplasmic redistribution of SENP3 where it co-localizes with FIH1 (Figure 5A middle panels). Interestingly, overexpression of FIH1_ΔK75Y_ prevented FIH1-SENP3 association due to limited nuclear translocation of FIH1 (Figure 5A bottom panels), suggesting that SUMOylation at K75 of FIH1 is a prerequisite for its translocation from cytoplasm to nucleus and subsequent nuclear deSUMOylation by SENP3.

### FIH1 is subject to active SUMOylation/deSUMOylation in early placental development

We next investigated the physiological consequences of FIH1 SUMOylation in the developing placenta. Immunoprecipitation of FIH1 followed by Western Blotting for SUMO2/3 showed increased FIH1-SUMO2/3 association early in gestation (6-9 weeks), coinciding with lower FIH1 abundance during this gestational window (Figure 4D). In contrast, at a time when FIH1 expression was maximal (i.e. 12 weeks), FIH1 SUMOylation was reduced while its deSUMOylation by SENP3, as indicated by FIH1-SENP3 binding, was increased (Figure 4D). Immunofluorescence staining for SUMO2/3 in sections from 5 weeks placenta showed positive nuclear SUMO2/3 signal in subsets of cytotrophoblast cells (Figure 5B) similar to that found for FIH during the same gestational time point (Figure 1B). Interestingly SENP3 expression localized to nuclei of both cytotrophoblasts and syncytiotrophoblasts (Figure 5B).

One of the consequences of SUMOylation is the recruitment of ubiquitin ligases that subsequently target proteins for proteasomal degradation [35]. Immunoprecipitation analysis of FIH1-ubiquitin complexes revealed association between FIH1 and ubiquitin early on in gestation that peaked at 10-11weeks (Figure 4E). Besides ubiquitin, it has been reported that there are specific preferential associations between unique ubiquitin ligases and specific SUMO proteins. In particular, SUMO3 has been described to interact with the ubiquitin ligase, Ring Finger Protein 4 (RNF4) [23]. Hence, we examined RNF4 protein abundance in early placentation. Indeed, RNF4 protein levels were elevated early in development (7-9 weeks) (Figure 4F). Immunofluorescence for RNF4 and FIH1 in JEG-3 cells at 3% O_2_ showed that in comparison to empty vector controls, FIH1_WT_ overexpression induced the accumulation of RNF4 to the peri-nuclear region where it associates with FIH1 (Figure 5C middle panel). In contrast, upon overexpression of ΔFIH1_K75_, RNF4 failed to accumulate in the peri-nuclear region and remained predominantly diffused across the cytoplasm, with minimal association to FIH1 (Figure 5C right panel).

### FIH1 SUMOylation regulation is disrupted in preeclampsia

**Figure 6:**
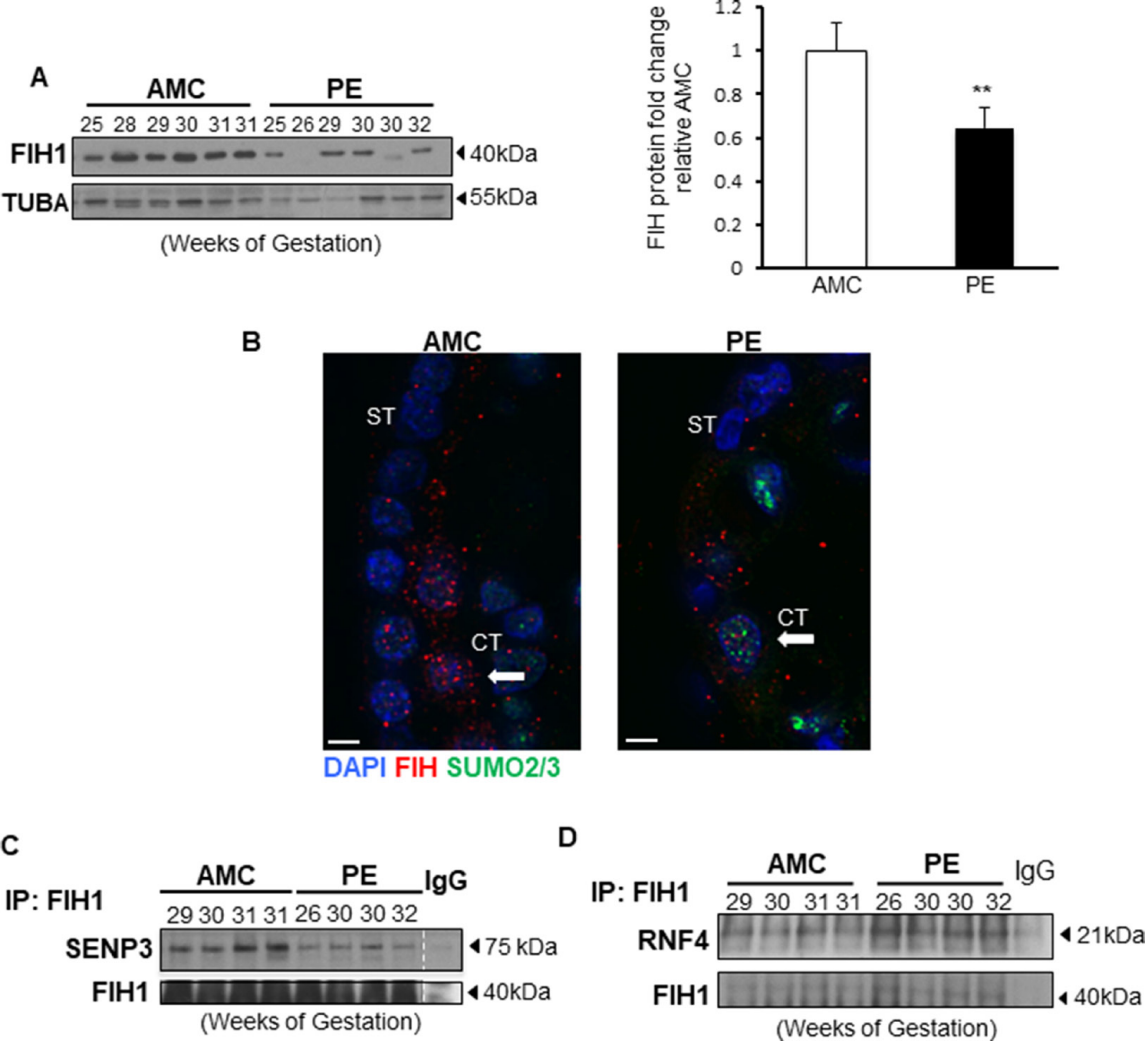
FIH1 deSUMOylation is impaired in PE leading to decreased FIH1 levels (**A**) Representative Western Blots for FIH1 in preeclamptic placentae (PE) and in normotensive age-matched controls (AMC), and respective densitometric analysis (right panel) *p* < 0.01 (*n* = 6 AMC, *n* = 7 PE). (**B**) Immunofluorescence analysis of FIH1 (red) and SUMO2/3 (green) in in PE and AMC placental tissue. Nuclei were counterstained with DAPI (blue). White bars represent 25 µm. (**C**) Representative Western Blots for SENP3 and FIH1 after FIH1 immunoprecipitation in PE (*n* = 9) and AMC (*n* = 9) tissue. (**D**) Representative Western Blots for RNF4 and FIH1 after FIH1 immunoprecipitation in PE and AMC tissue.

In line with our previous reports showing altered FIH1 mRNA and protein levels in PE placentae [1], herein we confirmed by Western Blotting that FIH1 expression is indeed significantly reduced in PE when compared to AMC (Figure 6A). In line with the Western Blotting data, immunofluorescence analysis showed that in placental sections from AMC, FIH1 is expressed within the trophoblast cell layers, whereas in PE, its signal is decreased (Figure 6B). Additionally, the presence of nuclear SUMO2/3 was detected in a subset of cytotrophoblast cells in sections from PE placentae (Figure 6B). Interestingly, immunoprecipitation of FIH1 followed by Western Blot analysis for SENP3 showed a marked disruption of FIH1 deSUMOylation by SENP3 in preeclampsia, relative to AMC placentae (Figure 6C). In addition, immunoprecipitation analysis of FIH1-RNF4 in PE show increased association of this ubiquitin ligase to FIH1 in preeclamptic placentae (Figure 6D).

## DISCUSSION

FIH1 studies have largely focussed on the role of this hydroxylase as a repressor of HIF1A transcriptional activity. However, more recently, the FIH1 field has thrived with discovery of novel targets and its diverse functions in signalling, apoptosis, migration and wound healing [36–39]. Despite these advances, the mechanisms regulating FIH1 stability remain elusive. In the present study, we show that placental FIH1 protein abundance directly correlates with physiological changes in oxygen tension during early gestation and that this is accompanied by FIH1 redistribution between nucleus and cytoplasm in trophoblast cells. Importantly, we demonstrate for the first time that FIH1 is subject to SUMOylation in the human placenta, and that this event is heightened in hypoxic conditions and in preeclampsia. Moreover, we show that SUMO3-mediated SUMOylation of FIH1 targets it for ubiquitination and proteasomal degradation.

When FIH1 was initially characterized, it was described as a predominantly cytoplasmic protein with low presence in the nuclei of osteosarcoma cells [40]. However, given its relatively low molecular weight, FIH1 should be able to diffuse freely across the nuclear membrane. Two photon confocal scanning microscopy subsequently showed that FIH1 was excluded from the nucleus, which led to the assumption of the existence of a shuttling mechanism that favourably maintained FIH1 in the cytoplasm [40]. However, nuclear localization of FIH1 has been reported in breast cancer cells [41] and in the rat nucleus pulposus [37]. Our immunofluorescence data using human JEG-3 cells maintained in low oxygen and human placental tissue sections from early gestation conclusively show that FIH1 localizes primarily to the nucleus in *in vivo* and *in vitro* hypoxic conditions, while it has a cytoplasmic appearance with increasing oxygenation, supporting the notion of FIH1 sub-cellular shuttling.

PHD enzymes are strictly regulated by oxygen and targeted for degradation in hypoxia [14, 42] [1]. In general, there is very little consensus as to the impact of cellular oxygen on FIH1 expression. Work done in RCC4 cells suggests that FIH1 is not directly regulated by varying oxygen tension [43]. Previous work from our laboratory has shown that *FIH1* mRNA levels remain unchanged with variations in oxygen tension in an *ex vivo* model of human placental villous explants [13]. In contrast, Hirose *et al* [37] reported that both FIH1 mRNA and protein were decreased in hypoxia in rat nucleus pulposus cells. There is also an ongoing debate on whether FIH1 levels are regulated via the MINT3 (Amyloid beta A4 precursor protein-binding family A member 3) and MT1-MMP (Membrane type 1 matrix metalloprotease) axis as FIH1 is sequestered to the plasma membrane by MINT3 in rat macrophages and nucleus pulposus cells in hypoxia [37, 44, 45]. Another interesting perspective on the oxygen-dependent regulation of FIH1 comes from work on FIH1 affinity for ARDs (Ankyrin Repeat Domains), a tertiary protein structure that mediates protein-protein interactions [46]. Under hypoxic conditions, FIH1 affinity is greater for ARDs than for HIF1A, leading to impaired HIF1A transcriptional inhibition [47]. FIH1 is an important element to the adaptation of chronic hypoxia, and our previous work using an *in vivo* system of chronic hypoxia, exemplified by high altitude pregnancies, revealed an increase in FIH1 protein levels, as part of a physiological adaptation to low oxygen [48]. In line with that observation, herein, we show an oxygen-dependent adaptation of FIH1 in the developing placenta.

Mechanisms regulating *FIH1* mRNA expression have predominantly been investigated in the cancer field; however, the mechanisms regulating FIH1 protein stability remain elusive. In contrast to FIH1, studies have conclusively demonstrated that the HIF1A regulatory PHDs are targeted for proteasomal degradation by the E3 ubiquitin ligases SIAH1a/2, particularly in hypoxia [14]; and we have shown that in preeclamptic placentae, characterised by hypoxia, degradation of PHDs by SIAHs is impaired [1]. Herein, we identify for the first time, that FIH1 protein is a target of SUMO3-mediated SUMOylation in both JEG-3 cells and human placental tissue. Despite the fact that the consequences of SUMOylation are target-dependent, one of the canonical effects of SUMOylation is subsequent targeting for proteasomal degradation via ubiquitin ligase recruitment [17]. In accordance with this, our data show that FIH1 SUMOylation results in its proteasomal degradation, and this aids to the maintenance of FIH1 and HIF1A homeostasis during placental development.

Complementary to SUMOylation, protein deSUMOylation is carried out by Sentrin specific proteases (SENPs) that are specialized in removing SUMO groups from their target proteins [20]. The cycle of SUMOylation and deSUMOylation is an extremely rapid and dynamic event. In this study, we show that SENP3 is indeed an important component of SUMO2/3-dependent FIH1 regulation and is involved in maintaining FIH1 homeostasis.

The E3 ubiquitin ligase, RNF4 has been shown to interact with proteins that are polySUMO conjugates for SUMO2/3-mediated SUMOylation [25, 49], with diverse downstream consequences [25, 50]. In the present study, we show that FIH1 binds to the SIM domains comprising RNF4 in JEG-3 cells giving credence to this ligase in controlling FIH1 protein stability. In line with this finding, we report that RNF4 protein levels are elevated at 7-9 weeks of gestation and inversely correlate with those of FIH1. In support of the idea of RNF4 acting on FIH1-SUMO conjugate, RNF4 has been described to relocate into areas in the cell where SUMOylation events occur [51] and cases of nuclear exclusion have been reported [52]. The presence of FIH1 and RNF4 in the peri-nuclear region in hypoxia suggests that FIH1 likely shuttles from the nucleus to the cytoplasm upon SUMOylation by SUMO2/3, a process that appears to be critical for its cytoplasmic degradation by the ubiquitin-proteasome system. Future studies may benefit from unravelling the precise mechanisms regulating FIH1 nuclear-cytoplasmic shuttling, particularly in the context of hypoxia.

In addition to uncovering mechanisms of FIH1 regulation in placental development, we report for the first time that FIH1 deSUMOylation is altered in PE, a finding that has important implications for FIH1 function in this pathology [53]. This is in line with our previous reports of enhanced deSUMOylation of HIF1A in PE, thereby contributing to its stabilization in this pathology [32]. Similarly, despite the overall increase in SENP3 expression in PE [32], herein we show that the association between FIH1 and SENP3 is completely abrogated, likely contributing to decreased FIH1 expression found in PE. Further studies are warranted to uncover the underlying molecular mechanisms responsible for the disrupted deSUMOylation events. In addition, the increased association with RNF4 is suggestive of a potential increased ubiquitination and proteasomal degradation of FIH1, supporting the hypothesis of active FIH1 degradation in PE. Furthermore, our findings are also in line with increased HIF1A transcriptional activity in PE, presumably due to lack of FIH1-mediated repression.

**Figure 7:**
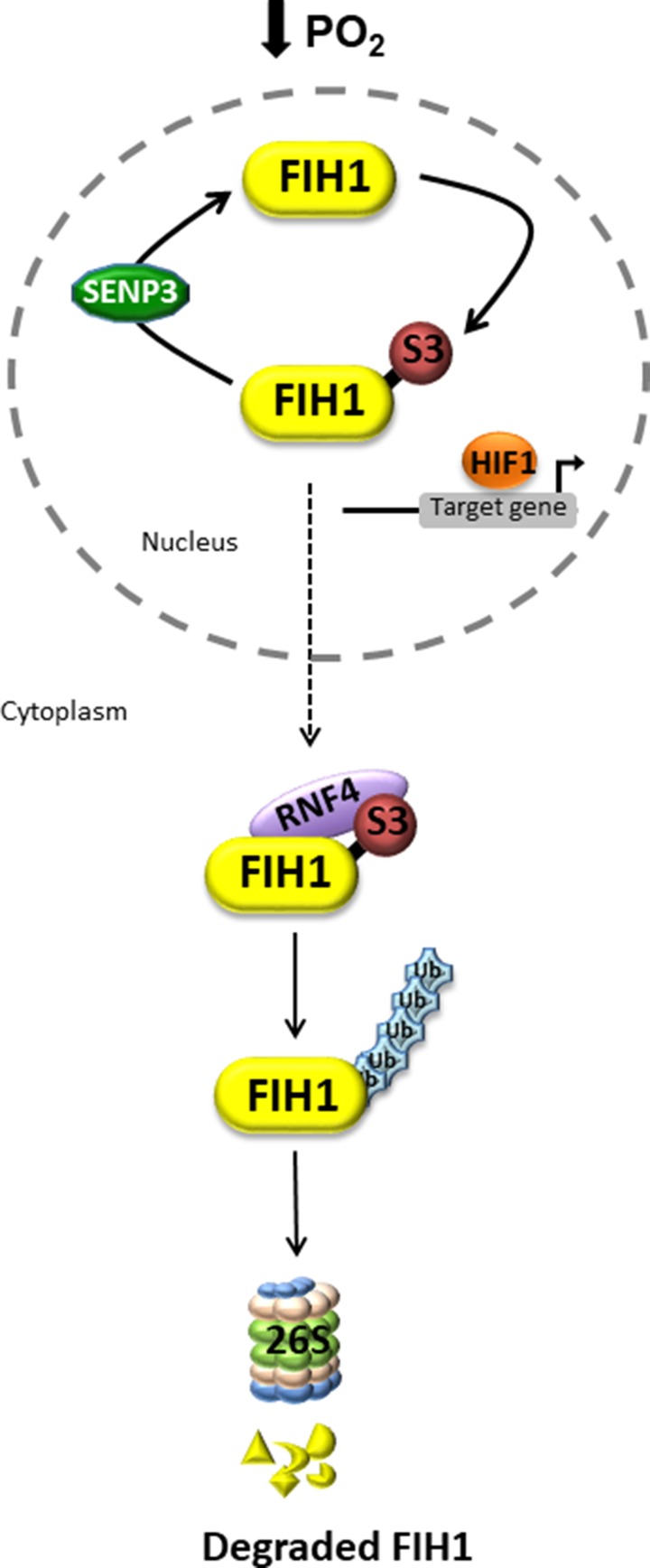
Putative model of dynamic FIH1 regulation by SUMOylation and deSUMOylation In low oxygen, nuclear FIH1 is targeted for SUMOylation by SUMO3, a process that is reversed by deSUMOylation via SENP3. This maintains HIF1A transcriptional activity. The consequence of FIH1 SUMOylation is the cytoplasmic recruitment of the ubiquitin ligase RNF4, which in turn promotes FIH1 ubiquitination and subsequent proteasomal degradation. In PE, lack of FIH1 deSUMOylation leads to accelerated FIH1 degradation thereby maintaining HIF1A transcriptional activity.

In summary, the present study uncovers a novel oxygen-dependent mode of regulation of FIH1 protein stability. In hypoxia, FIH1 is SUMOylated by SUMO3, possibly facilitating the recruitment of the ubiquitin ligase, RNF4, which in turn may target it for proteasomal degradation (Figure 7). In this way, in the developing placenta, FIH1 and consequently, HIF1A homeostasis is tightly maintained through physiological variations in oxygen tension. In PE, FIH1 evades deSUMOylation by SENP3 and SUMOylated FIH1 may be then targeted for RNF4-mediated degradation.

## MATERIALS AND METHODS

### Tissue collection

Placental tissue was collected following informed consent from pregnant females, according to the Ethics Guidelines of the University of Toronto’s Faculty of Medicine and Mount Sinai Hospital, Toronto. Human placental tissues from early gestation (5-14 weeks; *n* = 46) were obtained from elective terminations of pregnancies by dilation and curettage or suction evacuation, while term placental tissue was collected from deliveries at Mount Sinai Hospital, Toronto. Placentae were also collected from pregnancies complicated by PE and normotensive age-matched controls (AMC). The diagnosis of PE was made according to the ACOG criteria [3]. Control placental samples were obtained from women who did not exhibit signs of preeclampsia or other placental disease. The preterm deliveries were caused by incompetent cervix, spontaneous premature membrane rupture or spontaneous preterm deliveries without discernible cause. All collections were performed by the research center for women and infant health (RCWIH) Biobank from Mount Sinai Hospital. Standardized collection procedures include collection of placentae within 10 min of delivery (vaginal or C-section). The standard operating procedures can be found online on the RCWIH website (http://biobank.lunenfeld.ca). The clinical parameters of the patients enrolled in this study are reported in Table 1.

**Table 1:** Clinical parameters of participants

Conditions	PE (*n =* 19)	AMC (*n =* 15)
Mean GA (wks)	29.6 ± 0.83	29.3 ± 0.76
Blood pressure		
Systolic	172.7 ± 3.4	110.2 ± 4.4
Diastolic	105.9 ± 3.1	68.2 ± 2.12
Fetal Weight (g)	1238.6 ± 131.9	1497.1 ± 186.9
Mode of Delivery (%)	CS: 77.8	CS: 37.5
	VD: 22.2	VD: 62.5

Data are represented as mean ± standard deviation

PE, preeclampsia; AMC, preterm age-matched control

GA: gestational age

CS: Caesarian section; VD: Vaginal delivery.

### Cell culture

JEG-3 human choriocarcinoma cells (ATCC, Manassas, VA, USA; authenticated by Short Tandem Repeat genotyping) were cultured in Eagle’s minimal essential medium (EMEM) supplemented with 10% fetal bovine serum (FBS) and 10,000 units/ml of penicillin/streptomycin. Cells were cultured either in ambient (21% O_2_) air or in an atmosphere of 3% O_2_ (92% N_2,_ 5% CO_2_) for 24 hours at 37°C.

### Proteasome inhibition assay

JEG-3 cells, maintained at either ambient or hypoxic conditions, were treated with 5 µM MG-132 (SIGMA-Aldrich, Saint-Louis, USA), a selective inhibitor of the 26S proteasome, for 24 hours. Cells were then collected on ice in RIPA buffer and stored at -20°C.

### Plasmids and transfections

pcDNA-FIH1 plasmid construct was a gift from Eric Metzen (Addgene plasmid ^#^21399) and FIH1 mutant plasmids ΔFIH1_K75Y_ and ΔFIH1_K75Y/K177Y_ were created by introduction of missense mutations. pcDNA3.1 (V790-20 Invitrogen™, Life Science Inc., Burlington, ON) was used as an empty vector. Myc-SUMO1, Myc-SUMO2 and Myc-SUMO3 were generous gifts of Dr. Paul Sadowski (University of Toronto). HRE-luciferase was a gift from Navdeep Chandel (Addgene plasmid ^#^26731). Promoter-less Renilla-Luciferase in a pHRL-TK vector (Genbank AF362545) was used as non-responsive internal control in the HRE-luciferase assay. Transfections were performed using jetPRIME^®^ reagent (Polyplus transfection, Illkirch, FR). One µg of plasmid was combined with 200µL of jetPRIME^®^ buffer and 4µL of Jetprime^®^ reagent per reaction. Following incubation for 20 minutes at room temperature, 200 µL of the mix was added per well in a 6-well plate to JEG-3 cells. Following incubation for 48 hours in ambient (21% O_2_) or hypoxic (3% O_2_) conditions, cells were collected and processed for Western Blotting.

### Antibodies

Primary antibodies purchased from Santa Cruz Biotechnology (Santa Cruz, USA) were goat polyclonal FIH1 (N-18) (WB [1:500] IP [1:500] IF [1:1000]), mouse polyclonal SUMO1 (D-11) (WB [1:500]), goat polyclonal TUBA (P-16) (WB [1:1000]) and goat polyclonal ACTB (I-19) (WB [1:2000]). Primary antibodies purchased from AbCam (Cambridge, MA, USA) were rabbit monoclonal Sumo 2/3 (EPR4602) (WB [1:1000] IF [1:200]), rabbit polyclonal RNF4 (Ab166680) (WB [1:750] IF [1:400]), rabbit polyclonal FIH1 (Ab 12289) (WB [1:1000]) and mouse monoclonal CDH1 (Ab1416) (IF [1:100]). Rabbit monoclonal SENP3 (D20A10) (WB [1:500] IF [1:400]) antibody was purchased from Cell Signalling Technology (Beverly, MA, USA). Secondary horseradish-peroxidase conjugated donkey anti-goat, goat anti-rabbit and goat anti-mouse antibodies (WB [1:2000]) were from Santa Cruz Biotechnology. Alexa Fluor^®^ 488 donkey anti-goat/-rabbit/-mouse and Alexa Fluor^®^ 594 donkey anti-goat/-rabbit/-mouse [IF 1:200] were from Invitrogen (Carlsbad, CA, USA).

### Immunoprecipitation and Western blotting

Protein samples (ranging from 200 to 500 µg) were diluted in RIPA buffer, to a final concentration of 1 µg/µL and pre-cleared using Protein G agarose beads (10 µL) for 1 hour at 4°C. Samples were centrifuged at 200xg and supernatants were incubated with FIH1 antibody overnight at 4°C. The negative control consisted of non-immune goat IgG (concentration of 1µg per 100 µg of protein lysate). ProteinG-agarose beads (30 µL) were then added and samples were incubated for another 1 hour at 4°C. Immune complexes were then spun down (5 min at 500 g), rinsed with ice-cold PBS and resuspended 10% glycerol, 2% SDS, 5% β-mercaptoethanol, 0.0025 bromophenol blue, 0.06M Tris base. Aliquots of resuspended protein samples (50 μg) were then subjected to 12% SDS-PAGE, transferred to polyvinylidene fluoride (PVDF) membrane and immunoblotted as previously described [54]. Quantification was performed by normalization of the protein of interest to α-tubulin (TUBA) or β-actin (ACTB).

### Poly-SUMOylated protein purification

JEG-3 whole cell lysate was enriched in polySUMOylated proteins following guidelines from PolySUMO Capture & Release kit (UBPBio, Aurora, CO, USA). In brief, JEG-3 cells cultured in 3% oxygen were collected in lysis buffer complemented with Iodoacetamide (IAA, 200 mM), followed by sonication and ultracentrifugation (45 min, 90000 g) steps. PolySUMOylated protein were purified using SIM domains of RNF4 protein to capture targeted proteins. Elution was performed using competitive peptide for SIM domains [55].

### Immunofluorescence staining

Immunofluorescence was performed as previously described [56]. JEG-3 cells were plated on glass cover slips, grown to 70–80% confluency and then fixed in 10% paraformaldehyde for 15 minutes at room temperature. Cells were permeabilized with 0.2% triton X-100 (Bishop Canada Inc, Burlington, ON, Canada) and blocked in 5% horse serum and incubated with primary antibody diluted in antibody dilutant (0.4% sodium azide, 0.625% gelatine in PBS) containing 5% horse serum at 4°C overnight. Cells were then incubated with secondary antibody, stained with DAPI (4’,6-diamino-2-phenylindole) and mounted on slides. In case of placental tissue, sections of human placenta were permeabilized with 0.2% Triton X100 in PBS prior to antigen retrieval in 10 mM sodium citrate buffer solution. Black Sudan (0.3%) in 70% ethanol was used to quench endogenous fluorescence. Blocking was performed using 5% horse serum diluted in PBS, and sections were incubated with primary antibody overnight at 4°C in 5% horse serum. Sections were then incubated with secondary fluorescent-conjugated Alexa Fluor antibody, counterstained with DAPI and mounted with coverslips. Images were viewed and captured using the DeltaVision Deconvolution microscopy (Applied Precision, LLC, Issaquah, WA, USA).

### Gene reporter assay

HEK-293 cells (Source) were plated at a density of 2 × 10^5^cells/well, cultured for 24 hours in ambient air and then transiently transfected according to the above described method with the HRE-responsive reporter construct, HRE-Luciferase, in combination with empty vector pcDNA3.1 or pcDNA-FIH1_WT_ with or without Myc-SUMO3. The HRE-luciferase plasmid was co-transfected under 3% O_2_ in the presence of pcDNA-FIH1_WT_ with or without Myc-SUMO3. Cells maintained at 21% O_2_ following transfection with empty vector pcDNA3.1 were also used as control. Renilla luciferase was used as a non-HIF1A-responsive plasmid for normalizing transfection efficiencies and monitoring cell viability.. The total amount of plasmid DNA was normalized to 1.0 mg/well using empty pcDNA3.1 vector. After 24 hours, cells were cultured 3% and 21 % O_2_ for 6 hours at 37°C. Cells were harvested according to the manufacturer protocol in lysis buffer and lysates were processed using Dual-Luciferase^®^ Reporter Assay System kit (Promega, Madison, WI). Luciferase activity was measured using a Micro-Lumat Plus LB96V luminometer (EG&G Berthold; Oak Ridge, CA). HRE-Luciferase activity was normalized to constitutive TK driven promoter Renilla-luciferase. Experiments were repeated four times in triplicates.

### Statistical analysis

Statistical analyses were performed using GraphPad Prism 5 software (San Diego, CA, USA) and significance was established using a parametric Student’s *t*-test or one-way analysis of variance (ANOVA) with post-hoc Dunnett or Newman-Keuls test, where applicable. Statistical significance was defined as *p* < 0.05 and all data are represented as mean ± SEM of at least three separate experiments.

## SUPPLEMENTARY MATERIALS FIGURE



## Abbreviations

ACOG: The American Congress of Obstetricians and Gynaecologist
ACTB: Actin beta
ARD: Ankyrin repeated domain
AMC: Age-matched control
CBP/P300: Creb-binding protein/P300
CDH1: E-cadherin
CT: Cytotrophoblast cell
DAPI: 4’,6-diamino-2-phenylindole
EV: Empty vector
ECL: enhanced chemiluminescence
EMEM: Eagle minimum essential media
FBS: Fetal bovine serum
FIH1: Factor Inhibiting HIF1A
HEK-293: Human embryonic kidney cells
HIF1A: Hypoxia Inducible Factor 1 alpha
HIF1B: Hypoxia Inducible Factor 1 beta
HRE: Hypoxia responsive element
HRP: horseradish peroxidase
IAA: Iodoacetamide
IP: Immunoprecipitation
IF: Immunofluorescence
IgG: Immunoglobulin G
JmjC: Jumonji C domain
kDa: kilodalton
O_2_: Oxygen
ODD: Oxygen dependent degradation domain
PE: Preeclampsia
PVDF: Polyvinylidiene Fluoride
PBS: Phosphate buffered saline
PHD: Prolyl hydroxylase Domain
PML: Promyelotic Leukemia
RCC4: Renal cell carcinoma 4
RIPA: Radioimmunoprecipitation assay
RNF4: RING finger protein 4
ROS: Reactive oxygen species
SDS-PAGE: Sodium dodecyl sulfate; polyacrylamide gel electrophoresis
SIAH: Seven in absentia homolog
ST: Scyncytiotrophoblast cell
STDEV: Standard Deviation
STUBL: SUMO-targeted ubiquitin ligase
SUMO: Small ubiquitin like modifier
SENP: Sentrin Specific protease
TBST: Tris buffered saline-tween
TUBA: Tubulin alpha
VHL: von Hippel Lindau factor
WB: Western blot
WT: Wild type
