# Supplementary material for: Factor inhibiting HIF1–A novel target of SUMOylation in the human placenta
Source: Oncotarget. 2017 Dec 7;8(69):114002–18. doi: 10.18632/oncotarget.23113 (PMC5768381; doi:10.18632/oncotarget.23113)
Supplement: Supplementary file 1 [file oncotarget-08-114002-s001.pdf]

Factor inhibiting HIF1–A novel target of SUMOylation in the human placenta

SUPPLEMENTARY MATERIALS

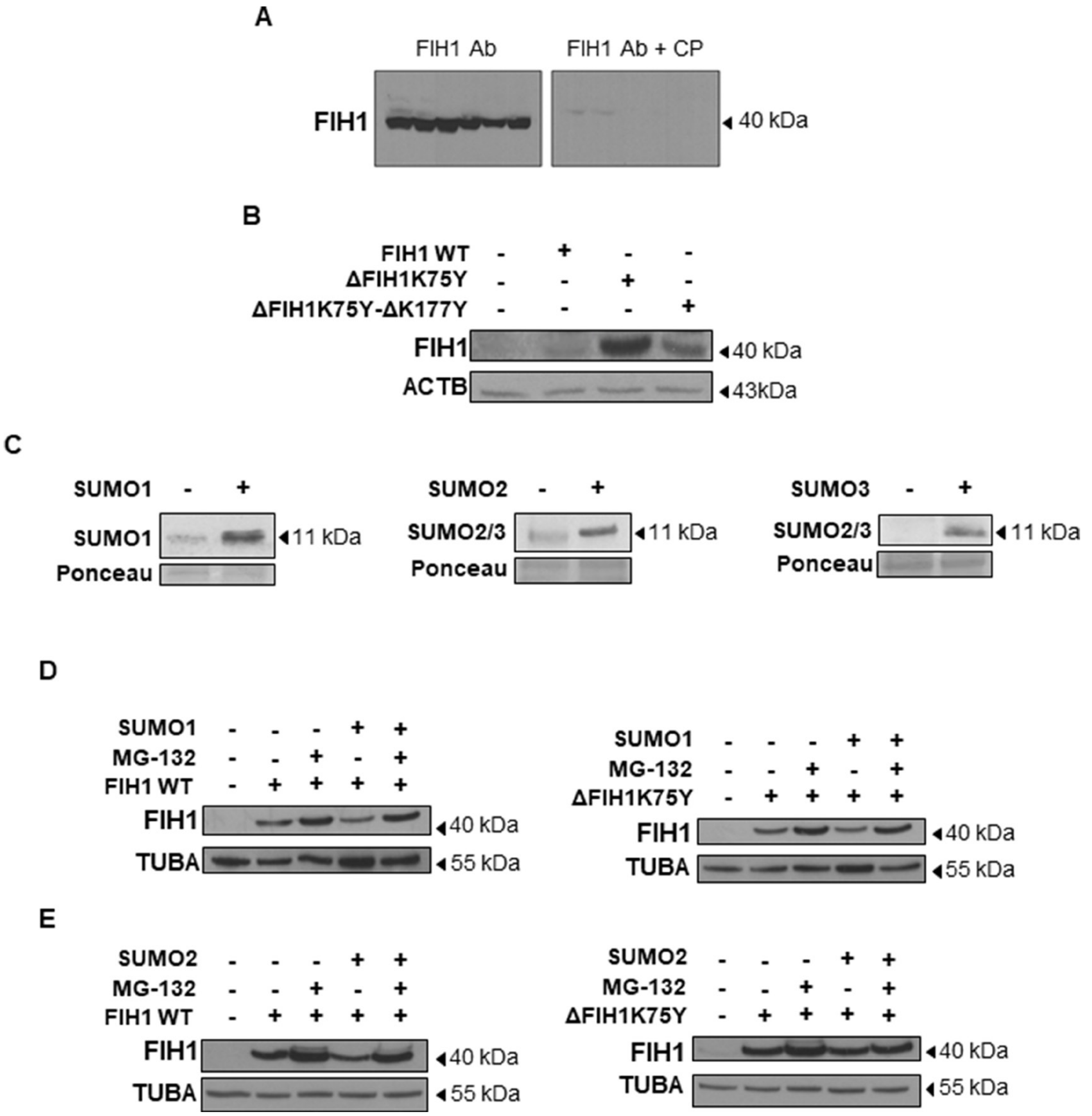

Supplementary Figure 1: (**A**) Western Blot for FIH1 in first trimester placental tissue probed with FIH1 antibody (left) and FIH1 antibody + competing peptide (right). (**B**) Representative Western Blots of FIH1 following FIH1 overexpression using plasmids construct containing either FIH1 WT, FIH1 ΔK75Y, or FIH1 ΔK75Y ΔK177Y. FIH1 antibody specifically recognized the C-terminal part of FIH1 protein. (**C**) Western Blot for SUMO1 and SUMO2/3 in JEG-3 cells following overexpression of SUMO1, SUMO2 or SUMO3 constructs. (**D**) Representative Western Blots for FIH1 in JEG-3 following overexpression of FIH1 WT (left panel) or FIH1 ΔK75Y (right panel) and SUMO1 protein in the presence and absence of the MG-132. (**E**) Representative Western Blots for FIH1 in JEG-3 following overexpression of FIH1 WT (left panel) or FIH1 ΔK75Y (right panel) and SUMO2 protein in the presence and absence of the MG-132.
